# Analysis of copy number variations of *WNT4* gene in a Chinese population with Müllerian anomalies

**DOI:** 10.1186/s13023-021-01888-0

**Published:** 2021-06-07

**Authors:** Ying Zhu, Ruyi Wang, Yun Cheng, Yang Han, Tengyan Li, Yunxia Cao, Binbin Wang

**Affiliations:** 1 Reproductive Medicine Center, Department of Obstetrics and Gynecology, The First Affiliated Hospital of Anhui Medical University, 218 Jixi Road, Shushan, Hefei, 230022 China; 2 Anhui Province Key Laboratory of Reproductive Health and Genetics, Biopreservation and Artificial Organs, Hefei, China; 3 Anhui Provincial Engineering Research Center, Anhui Medical University, Hefei, People’s Republic of China; 4 Graduate School of Peking Union Medical College, Beijing, China; 5 Center for Genetics, National Research Institute for Family Planning, 12 Dahuisi Road, Haidian, Beijing, 100081 China

**Keywords:** Copy number variations, *WNT4*, Müllerian anomalies, Chinese population

## Abstract

**Background:**

To investigate the genetic contribution of copy number variations (CNVs) in Wingless-type MMTV integration site family, member 4 (*WNT4*), in a Chinese population with Müllerian anomalies (MA), copy number analysis of *WNT4* by Multiplex ligation‐dependent probe amplification (MLPA) was performed on 248 female patients. Some studies have shown that heterozygous missense mutation of *WNT4* can lead to MA. However, few studies on the relationship between *WNT4* CNVs and MA have been performed.

**Results:**

Among the 248 Chinese women affected by MA in this study, heterozygous deletion of *WNT4* was detected in a single patient.

**Conclusions:**

MLPA identified one heterozygous deletion in *WNT4* in a single female patient among 248 Chinese women affected by MA. This study firstly reports CNVs of *WNT4* in a large sample of MA patients from the Chinese population, which suggests that CNVs of *WNT4* cannot be excluded in the occurrence of MA. This provides a genetic basis for precise treatment in the future.

## Background

Müllerian anomalies (MA) are among the most common diseases in gynecology and obstetrics. They include various malformations of fallopian tubes, vagina, cervix, and corpus uteri [1]. The estimated incidence of MA is 5.5% in the general population, but 24.5% in women with infertility and miscarriage [2, 3]. This disease seriously affects women’s reproductive function and causes a huge social burden.

According to guidance of the European Society of Human Reproduction and Embryology/European Society for Gynaecological Endoscopy (ESHRE/ESGE) in 2013 [4], Müllerian duct abnormalities can be classified into 36 different combinations as the main class for the uterine body (U) and subclasses for the cervix (C) and vagina (V). Compared to the classification method put forward by American Fertility Society, ESHRE/ESGE classified uterine morphology into 7 classes: U0, normal; U1, dysmorphic; U2, septate; U3,bicorporeal; U4, hemiuterus; U5, aplastic; U6, unclassified cases.

Different types of Müllerian duct anomalies have different effects on reproductive function. People with mild symptoms can have normal fertility, but most patients with uterine malformations exhibit serious effects such as primary amenorrhea, recurrent abortion, and infertility. Septate uterus is one of the milder symptoms in patients with uterine malformation, and the rate of live births from such patients is about 62.5%. After operation, this rate can be significantly increased [5].

To date, many genes related to MA have been found, including many genes of the Wingless-type MMTV integration site family (*WNT4*, *WNT5A*, *WANT7*, *WNT9B*) [6–10]. *WNT4* (OMIM: 603490), a transcriptional regulator, plays an important role in sex determination, nephrogenesis, and female reproductive tract development in the early embryo [6, 11–13]. *WNT4*^*−*/*−*^ female mice were reported to show phenotypes of sex reversal, Müllerian duct aplasia, hyperandrogenism, and follicle depletion [6, 14].

Many studies have also shown that the heterozygous missense mutation of *WNT4* can lead to MA and hyperandrogenism in humans [15–18]. However, few studies have been performed on the relationship between *WNT4* copy number variations (CNVs) and MA. Therefore, here we detected the copy number of *WNT4* in 248 female patients with MA.

## Materials and methods

### Participants

248 unrelated MA patients were recruited from the Reproductive Medicine Center of the First Affiliated Hospital of Anhui Medical University. All patients were Chinese, ranging in age from 22 to 48 years (29.32 ± 4.87). All patients showed a normal karyotype of 46, XX and had been diagnosed with abnormal Müllerian ducts using physical examination, ultrasonographic investigations, hysteroscopy and laparoscopy. The study protocol was approved by the Ethics Committee of the National Research Institute for Family Planning, and written informed consent was obtained from all participants.

### DNA extraction

Genomic DNA was extracted from the peripheral blood of 248 patients using the QIAamp DNA Blood Mini Kit (Qiagen, Hilden, Germany).

### MLPA assay

Three probes designed in-house targeted the region of the *WNT4* gene. The first probe covered exon 1 and exon 2, second probe covered exon 3 and exon 4, third probe covered exon 5. Fluorescence data on the PCR products analyzed by capillary electrophoresis were collected using an Applied Biosystems 3730xl DNA Analyzer, and the peak area data were output. Relative peak area of *WNT4* was compared with that of the reference gene. Dosage quotient (DQ) was used as the signal result of CNVs. The classification was as follows: normal (0.8–1.2), homozygous deletion (0), heterozygous deletion (0.4–0.65), and heterozygous duplication (1.3–1.65).

### Analysis of CNV pathogenicity

The pathogenicity of CNVs was evaluated in accordance with the CNV interpretation scoring metric from the American College of Medical Genetics and Genomics (ACMG) [19]. Online databases including the Database of Genomic Variants (DGV, http://dgv.tcag.ca/dgv/app/home/) and the Genome Aggregation Database (GnomAD, http://gnomad.broadinstitute.org/) were used to retrieve the frequency of CNVs. Public databases, including PubMed (https://pubmed.ncbi.nlm.nih.gov/) and Online Mendelian Inheritance in Man (OMIM, https://www.omim.org/), were used to evaluate the significance of CNVs of *WNT4*.

## Results

### MLPA results of MA patients

**Fig. 1:**
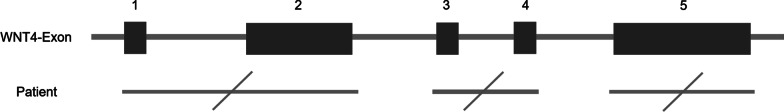
Copy number variations on *WNT4* exons. *WNT4* has 5 exons, one MA patient carried heterozygous deletion of *WNT4*. Gray slash symbols deletion

One deletion was identified in *WNT4* in a single female patient among the 248 Chinese women affected by MA in this study (Fig. 1). The DQ values of three probe recognition sequences were all between 0.4 and 0.65, reflecting a heterozygous deletion of *WNT4* (Table 1).

**Table 1:** CNVs of *WNT4* gene and clinical features in congenital uterine anomaly patients

Patient No	Copy number status	Dosage quotient	Age (years)	BMI (kg/m^2^)	FSH (IU/l)	LH (IU/l)	E2 (pmol/l)	P (nmol/l)	PRL (ng/ml)	T (nmol/l)
1	Heterozygous deletion of exons 1–5	0.51 (ex1–ex2) 0.61 (ex3–ex4) 0.45 (ex5)	28	20	6.06	3.23	75.5	1.43	15.54	0.4

*BMI* body mass index (normal range 18.5–23.9 kg/m^2^), *FSH* follicle stimulating hormone (normal range 2.5–10.2 mIU/ml), *LH* luteinizing hormone (normal range: 1.9–12.5 mIU/ml), *E2* estradiol (normal range 40–253 pmol/l), *P* progesterone (normal range 0.6–1.9 nmol/l), *PRL* prolactin (normal range 2.8–29.2 ng/ml); *T* testosterone (normal range 0.48–2.64 nmol/l)

### Clinical characteristics of the patient with CNVs

The patient with a single copy of *WNT4* was 28 years old, had regular menstruation, and exhibited normal levels of estradiol (E2) and testosterone (T). She suffered from secondary infertility, having been diagnosed with partial septate uterus but no renal abnormalities. She had undergone transcervical incision of septa (TCIS) in 2016 (see Table 1 for details).

### Online database analysis

No identical overlapping variant was found in the DGV database and GnomAD database. Therefore, this heterozygous whole deletion of *WNT4* was considered to be rare in the general population. In the OMIM database, the mutation of *WNT4* was reported to be associated with abnormal Müllerian ducts in women. The heterozygous deletion of *WNT4* was considered variant of uncertain significance (VUS) according to the ACMG guidelines (see Table 2 for details).

**Table 2:** CNV Interpretation Scoring Metric of ACMG

Patient no	Copy number status	Contain protein-coding elements (Score)^a^	Haploinsufficient genes^b^	Number of protein-coding RefSeq genes (Score)^c^	Analysis of public databases and literature (Score)^d^	Inheritance pattern for patient (Score)^e^	Total score	Classification^f^
1	Heterozygous deletion of *WNT4*	1A, Yes (0)	Skip	3A, 1 (0)	4E, reported phenotype is highly specific and relatively unique to *WNT4*. But the inheritance pattern of the variant is unknown (0.1)	5F, inheritance pattern is unknown (0)	0.1	VUS

^a^Gene type of *WNT4*: protein coding, Accession: NM_030761.5 (https://www.ncbi.nlm.nih.gov/)

^#^The haploinsufficiency evaluation of *WNT4* has not been established (https://dosage.clinicalgenome.org/), and *WNT4* was predicted to be tolerated to haploinsufficiency (gnomAD version 2.1.1 and Haploinsufficiency Predictions Version 3)

^c^We targeted the *WNT4* gene, so the number of protein-coding RefSeq genes is 1

^d,e^The blood of parents of the case is unavailable, so the inheritance pattern is unknown. But the patient has specific phenotype of MA and relatively unique to *WNT4* in women. (ClinVar, http://www.ncbi.nlm.nih.gov/clinvar, OMIM, https://www.omim.org/)

^f^Pathogenic: total score ≥ 0.99 or more points; likely pathogenic: 0.90 < total score < 0.98; VUS, variant of uncertain significance: − 0.89 < total score < 0.89; likely benign: − 0.98 < total score <  − 0.90; benign: total score ≤  − 0.99

## Discussion

*WNT4* has five exons and is located at 1p36.12 [genome coordinates (GRCh38): 1:2217307–22143980] (NCBI, https://www.ncbi.nlm.nih.gov/). It is a member of the WNT family of signaling molecules. The signaling protein encoded by this gene plays an important role in early embryonic sex determination, nephrogenesis, and female reproductive tract development [6, 13]. *WNT4*^*−*/*−*^ female mice have been reported to show the phenotypes of sex reversal, Müllerian duct aplasia, hyperandrogenism, and follicle depletion [6, 14]. *WNT4*^monomeric cherry/monomericcherry^ mice were shown to form Müllerian ducts in only 45% of cases [20].

The abnormal Müllerian ducts caused by *WNT4* mutation are rare, but are pathological. Four missense mutations of *WNT4* (p.L12P, p.E226G, p.R83C, p.A233) were found in four unrelated women with MA between 2004 and 2011 [15–18]. However, Chang et al. did not find any significant *WNT4* mutation in 189 Chinese women with MA in 2012, although they did not analyze the CNVs [21]. Meanwhile, we identified only one heterozygous deletion in *WNT4* in a single female patient among 248 Chinese women affected by MA. This shows the rarity of CNVs in MA patients. To our best knowledge, it is the first report of CNVin WNT4 in MA patients overall. And the deletion was assessed as VUS. Only missense variants have so far been associated with the phenotype, so that loss-of-function is not a known mechanism of disease. A VUS is not a benign or likely benign variant, the deletion we observed may possibly contributed to MA. So further evidence might accumulate future recognition of LOH as a pathogenic mechanism.

We also found that the abnormality of the Müllerian duct caused by missense mutation of *WNT4* differed from that caused by CNVs of *WNT4*. All of the women carrying the missense mutation showed Müllerian duct abnormalities (two patients showed absent vagina and uterus, while one 15-year-old patient showed uterine agenesis), hyperandrogenemia, and primary amenorrhea [15–18]. However, our patient with a septate uterus showed normal menstruation and testosterone (T). *WNT4* inhibits steroidogenic enzymes by upregulating *DAX1*. Steroidogenic enzymes [including 3b-hydroxysteroid dehydrogenase 2 (HSD3B2) and 17a-hydroxylase (CYP17A1)] are necessary for testosterone production. The four missense mutations found in previous studies have been proven to increase the levels of HSD3B2 and CYP17A1 in cells transfected with the mutant alone or in combination with the wild-type protein. It was concluded that mutation of *WNT4* showed a dominant negative effect [15–18]. The loss of heterozygosity (LOH) in the patient that we identified may only led to haploinsufficiency. Therefore, we believe that the phenotype caused by LOH of *WNT4* might be less severe than that caused by a dominant negative effect.

In addition, some rearrangements of other genes or regions found in MA patients should not be overlooked. Some of these regions have been shown to be clinically significant, such as 1q21.1, 7p14.3, 16p11.2, 17q12, and 22q11. In addition, some candidate genes have been proposed, including*LHX1*, *BBS9*, *HNF1B*, and *TBX6* [22–25].For example, Bernardini et al. identified deletion of 17q12 (including *LHX1*) in two cases with Mayer–Rokitansky–Kuster–Hauser (MRKH) syndrome [22]. Ledig et al. observed 22 CNVs (including in *LHX1*and *HNF1B*) at regions 1q21.1, 17q12, and 22q11.21 in 15 of 48 patients with MRKH [24]. Meanwhile, Gervasini et al. found *SHOX* duplications at chromosomal region Xp22 in 5 of 30 MRKH patients [26]. Thus, some other genes or regions should be noted in CNV analysis of MA patients.

## Conclusions

One heterozygous deletion in *WNT4* was identified by MLPA in a single female patient among 248 Chinese women affected by MA. This study reports CNVs of *WNT4* in a large sample of MA patients from the Chinese population for the first time. This suggests that CNVs of *WNT4* cannot be excluded in the occurrence of MA. This provides a genetic basis for precise treatment in the future.

## Data Availability

All data were generated or analyzed during this study are included in this article.

## Abbreviations

CNVs: Copy number variations
WNT4: Wingless-type MMTV integration site family, member 4
MA: Müllerian anomalies
MLPA: Multiplex ligation‐dependent probe amplification
ESHRE/ESGE: European Society of Human Reproduction and Embryology/European Society for Gynaecological Endoscopy
U: Uterine body
C: Cervix
V: Vagina
DQ: Dosage quotient
ACMG: American College of Medical Genetics and Genomics
DGV: Database of Genomic Variants
GnomAD: Genome Aggregation Database
OMIM: Online Mendelian Inheritance in Man
E2: Estradiol
T: Testosterone
TCIS: Transcervical incision of septa
HSD3B2: 3B-hydroxysteroid dehydrogenase 2
CYP17A1: 17A-hydroxylase
LOH: Loss of heterozygosity
MRKH: Mayer–Rokitansky–Kuster–Hauser
